# Effect of knee flexion angle on the direction and magnitude of tensile force in complete lateral meniscus radial tears: A porcine biomechanical study

**DOI:** 10.1002/jeo2.70465

**Published:** 2025-10-28

**Authors:** Kodai Hamaoka, Tomoaki Kamiya, Kousuke Shiwaku, Kazushi Horita, Yasutoshi Ikeda, Yohei Okada, Hidenori Otsubo, Makoto Emori, Atsushi Teramoto

**Affiliations:** ^1^ Department of Orthopaedic Surgery Sapporo Medical University School of Medicine Sapporo Hokkaido Japan; ^2^ Sapporo Sports Clinic Sapporo Japan

**Keywords:** biomechanics, lateral meniscal repair, radial tear, tensile force

## Abstract

**Purpose:**

The effect of the knee flexion angle on the direction and magnitude of tensile force on the meniscus during flexion and extension under a load is unknown. This study aimed to clarify this effect on repair sutures in a lateral meniscus radial tear of the midbody under knee‐joint conditions during flexion and extension.

**Methods:**

This study was performed using 10 porcine knees, a robotic system with six degrees of freedom and a load cell. Meniscal repairs were performed using horizontal sutures, with a single stitch between the central and peripheral regions at (1) the inner third position and (2) the outer third position, each meniscus sequentially. The suture of the posterior segment was drawn tangentially anteriorly, whereas the suture of the anterior segment was drawn tangentially posteriorly. The suture of the posterior segment was connected to an anterior load cell to measure the tensile force applied to the suture in the posterior direction. Subsequently, the suture of the anterior segment was connected to a posterior load cell to measure the tensile force applied in the anterior direction. A valgus load of 5 N m was applied for three cycles at 30°, 60° and 90° flexion.

**Results:**

The tensile forces exerted anteriorly on the repair sutures at the inner and outer sides in the anterior segment at 30°, 60° and 90° flexions were 13.7/13.3 N (inner/outer), 7.6/7.3 N and 3.4/3.5 N, respectively. The tensile forces exerted posteriorly on the repair sutures in the posterior segment at 30°, 60° and 90° flexions were 1.8/1.5, 10.6/9.7 and 16.2/16.4 N, respectively.

**Conclusions:**

Tensile force was exerted on the repair sutures of complete lateral meniscus radial tears of the midbody, anteriorly in the anterior segment at 30° flexion and posteriorly in the posterior segment at 90° flexion.

**Level of Evidence:**

N/A.

**Abbreviation**
LM: lateral meniscus

## INTRODUCTION

The knee meniscus is a C‐shaped fibrocartilaginous structure that preserves the normal function of the knee joint and plays an important role in load distribution, shock absorption, stability, lubrication and proprioception [5].

A systematic review and meta‐analysis [14] reported that modern meniscal repair has an overall failure rate of 19.5% at a minimum of 5 years post‐operatively. According to arthroscopy evaluation, the failure rate of lateral meniscus (LM) radial tears after six months was 39% among meniscal injuries [30]. There are several reasons that LM radial tears are crucial injuries. Initially, the LM covers a greater percentage of the lateral plateau than the medial meniscus, bearing the majority of the load on the lateral side of the joint [3]. Next, radial tears disrupt the hoop structure, regardless of the medial meniscus and LM [2]. Additionally, the tibial surface in the lateral compartment is convex [1]. Complete LM radial tears have been shown to be functionally equivalent to a total meniscectomy [15].

Biomechanical studies have already reported the negative effects of LM radial tears in terms of contact pressure and area, and resultant force [15, 16, 25]. In addition, in a porcine model, an LM with a radial tear that is 90% of the width of the midbody displaces primarily in the anteroposterior direction relative to the tibia under an axial load at 30° and 60° flexion [7]. In a complete LM radial tear, peripheral gaps remained even after meniscal repair using the inside‐out technique [19].

Most previous biomechanical studies on meniscal repair were performed under non‐physiological conditions, in which the meniscus was isolated from animal or human cadaveric knees [12, 13, 23]. These studies examined the influence of repair techniques and suture materials, mainly assessed by loading to failure. However, to test the efficacy of the suture construct, studies that simulate the natural loading conditions of the meniscus and the femur–tibia relationship are necessary, rather than studies on specimens isolated from the knee joint.

Regarding the relationship to the knee flexion angle, a previous study reported that the contact area of the tibiofemoral joint in the lateral compartment shifted posteriorly from extension to flexion [33]. This was based on the fact that the lateral femoral condyle demonstrates consistent posterior translation throughout knee flexion in normal knees [28]. Ohori et al. [16] reported that a compressive load on the tibiofemoral joint is applied mainly to the anterior segment of the LM in the extended position and to the posterior segment in the deeply flexed position. However, the effect of knee flexion angles on the direction and magnitude of the tensile force on repair sutures during flexion and extension under a valgus load remains unknown. To prevent reinjury after meniscal repair, careful post‐operative rehabilitation is required to avoid overstressing the sutures. Clinically, previous studies have demonstrated that a non‐weight‐bearing period and immobilization with a brace are necessary for post‐operative rehabilitation following meniscal repair of LM radial tears to prevent the failure of the meniscus repair at the repaired site [30, 31]. Thus, biomechanical information regarding the tensile force is important for post‐rehabilitation considerations. Moreover, understanding how tensile force occurs after meniscal repair under loading conditions may provide useful information for investigating biomechanically superior meniscal repair techniques and materials.

This study aimed to clarify the effects of knee flexion angles on the direction and magnitude of tensile force on repair sutures during flexion and extension under valgus load in a complete LM radial tear of the midbody using freshly frozen porcine knees, a robotic system and a load cell. We hypothesized that the direction and magnitude of the tensile force on the repair suture in a complete LM radial tear of the midbody would differ from the flexion angle under loading.

## METHODS

### Specimen preparation

Ten intact, freshly frozen porcine right knees were used in this study. None of the specimens showed osteoarthritic changes or ligamentous or meniscal injuries. The knees were frozen at −23°C and thawed at room temperature for 24 h. The femur and tibia were cut at least 15 cm above and below the joint line, respectively. The fibula was cut 5 cm below the proximal tibiofibular joint. The patella and soft tissues, including all muscles except the popliteus, were excised, whereas the ligaments, posterior capsule and meniscus were left intact. Both ends of the tibia and femur were fixed using acrylic resin (Ostron II; GC Australasia) poured into cylindrical moulds. The fibula was fixed in the anatomical position using an acrylic resin. The femoral and tibial cylinders were fixed with aluminium clamps and connected to the end effector of a robotic testing system (FRS‐2015; Technology Service, Chino) [4].

### Testing apparatus

A robotic testing system with a custom‐made manipulator with six degrees of freedom, equipped with a universal force–torque sensor (DELTA IP65, SI‐660‐60; ATI Industrial Automation) was used. This robotic system can simulate physiological knee joint motion, using the joint coordinate system developed by Grood and Suntay [6] as a reference. This system, which guides the displacement and force/torque applied to the knee joints, was controlled in real time using a LabView (version 1.0.0.0; National Instruments Corp.)‐based program running on a Windows PC (Microsoft).

The tensile force on the repair suture was measured using a load cell (LUX‐B‐200N‐ID; KYOWA Electronic Instruments Co.). The load cell could measure tension of up to 200 N with an accuracy of 0.3 N. The tensile forces generated were sampled at a frequency of 100 Hz.

### Testing protocol

The knee flexion angle was defined as 15° flexion with an extension moment of 1 N m because of the intrinsic lag in porcine knees. Each knee was subjected to three cycles of passive extension–flexion motion between 15° and 120° flexion for preconditioning. A valgus load of 5 N m was applied for three cycles at 30°, 60° and 90° flexion in the intact state [17, 19, 25].

**Figure 1:**
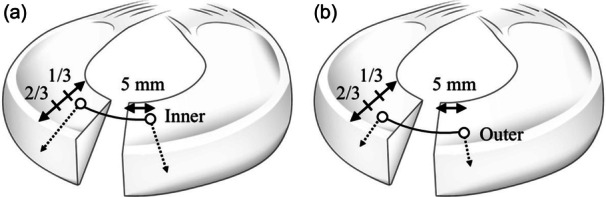
Schema of meniscal horizontal repair. (a) Single stitch between the central and peripheral regions at the inner third position. (b) Single stitch between the central and peripheral regions at the outer third position.

Subsequently, a complete LM radial tear was created at the midbody via arthroscopy using a No. 11 scalpel blade. Meniscal repair was performed using the inside‐out technique with No. 3 polyester sutures (Akiyama Seisakusyo). Meniscal repairs were performed using horizontal sutures, with a single stitch between the central and peripheral regions at (1) the inner third position and (2) the outer third position (Figure 1). The order was randomized.

**Figure 2:**
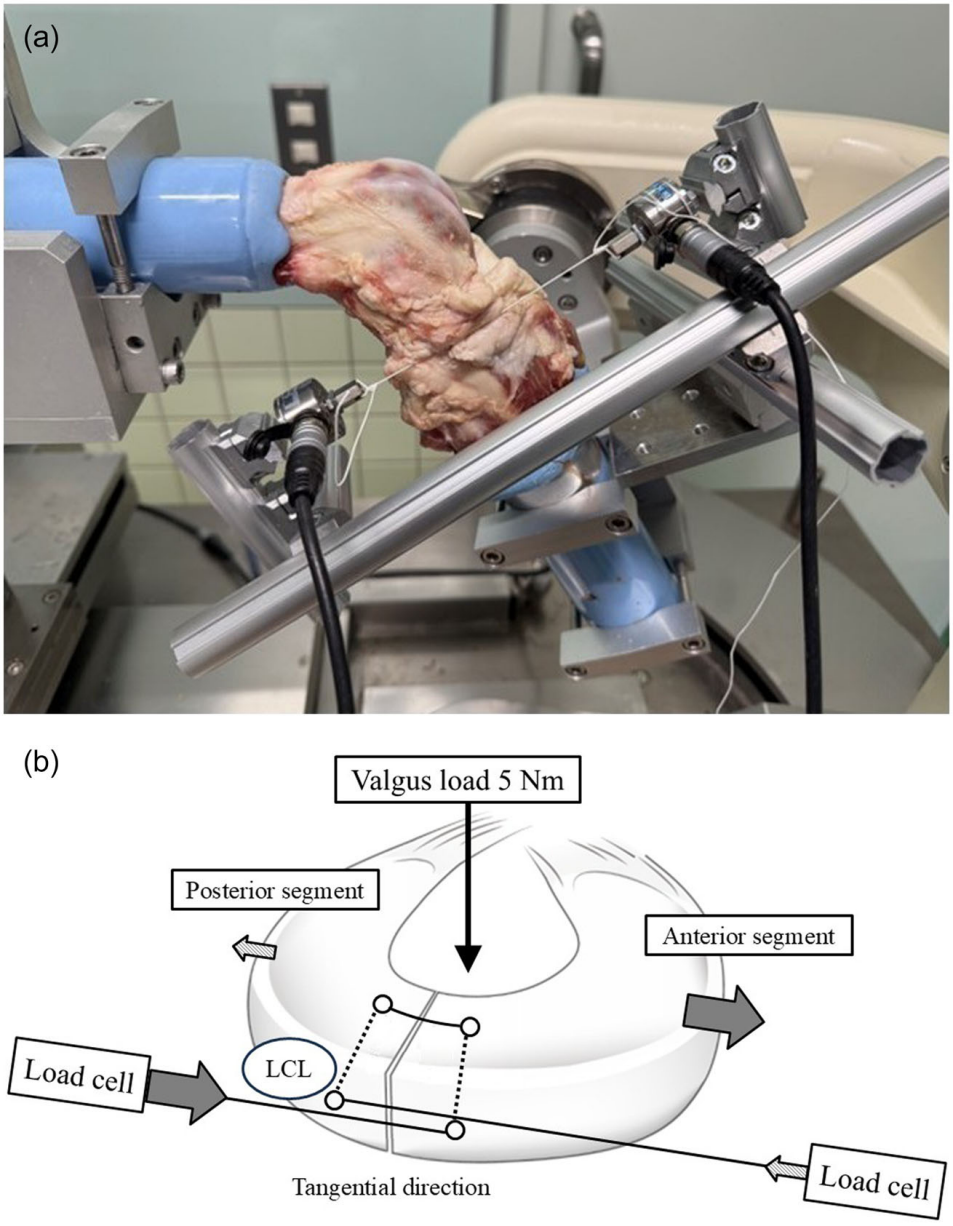
(a) Robotic testing system and two load cells to measure tensile force. (b) Schematic diagram of the tensile force measurement method. LCL, lateral collateral ligament.

Sutures were placed on the femoral side of the LM and 5 mm on either side of the tear. On the peripheral side of the meniscus, the suture of the posterior segment was drawn tangentially anteriorly, whereas that of the anterior segment was drawn tangentially posteriorly (Figure 2). The suture of the posterior segment was connected to a load cell on the anterior side to measure the tensile force applied in the posterior direction. Next, the suture of the anterior segment was connected to another load cell on the posterior side to measure the tensile force applied to the suture in the anterior direction. A pilot study investigated the effect of the initial tension on the peripheral gap under a valgus load, with the suture site capsule removed to achieve direct visualization. Because an initial tension of 2 N or more at each load cell did not lead to a peripheral gap at the LM under a 5 N m valgus load, the initial tension was fixed at 2 N or more (average 4.8 N) at each load cell in all porcine knees. Tensile force was calculated as the difference between the maximum tension under valgus load and the initial tension. Before each status and preconditioning test for each knee, a 5 N m valgus load was applied to the knee three times, at 30°, 60° and 90° flexion, to minimize the effects of creep. Subsequently, the tensile forces acting on the repair sutures were measured (Table 1). Based on a previous biomechanical study investigating LM radial tear using a robot simulator, a 5 N m valgus load was applied [17, 19, 25]. The experiments were repeated three times for each knee, and the median values were adopted.

**Table 1:** Acquisition of tensile force data.

Suture site	Directions for measurement	Applied load	Acquired data
Inner	Anterior/posterior	5 N m of valgus load (30°, 60°, 90°)	Tensile force
Outer			

### Study approval

The study protocol did not require approval from the Institutional Review Board of our institute, because all porcine knee specimens were obtained from a butcher.

### Statistical analysis

All statistical analyses were performed using EZR (version 1.61; Saitama Medical Center, Jichi Medical University), which is a graphical user interface for R (version 4.2.2; R Foundation for Statistical Computing). EZR is a modified version of the R Commander in which statistical functions frequently used in biostatistics have been added [10].

The tensile force on the repair suture under a valgus load in each translation direction was analyzed using two‐factor repeated‐measures analysis of variance to compare the flexion angles and stitching technique. A paired *t* test was used for post hoc analysis with Bonferroni correction.

Statistical significance was set at *p* < 0.05. The priori sample size calculation was performed using G‐power version 3.1.9.6, assuming a two‐factor repeated‐measures analysis of variance between six groups with an effect size of 0.5, a significant level set at 0.05 and a power of 0.8. The required sample size was calculated to be seven, and the study achieved a satisfactory level of statistical power.

## RESULTS

**Figure 3:**
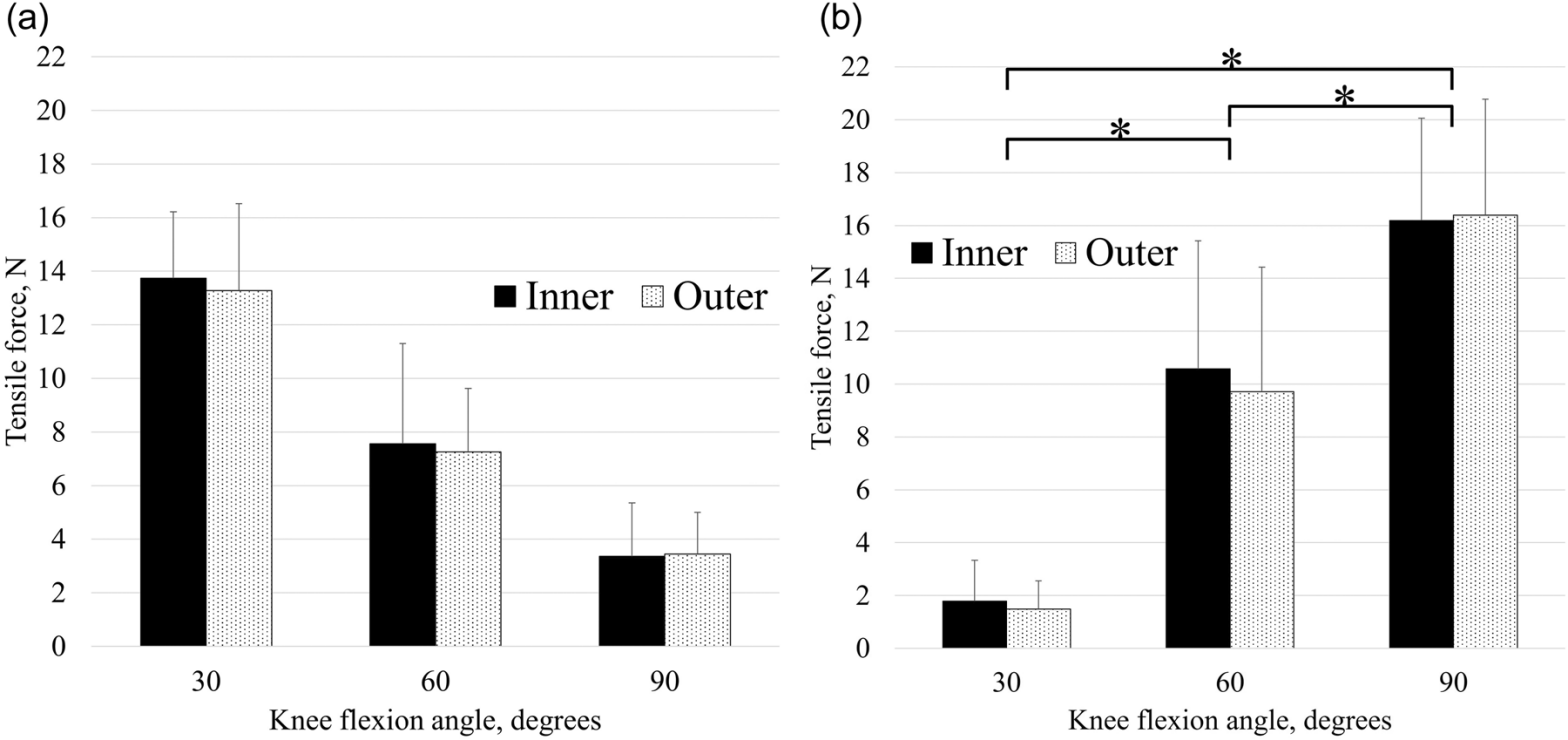
Influence of knee flexion angle on the tensile force exerted on repair sutures in anterior (a) and posterior (b) directions under a 5 N m valgus load. The forces were not significantly different between the inner and outer meniscal sides at 30°, 60° or 90° flexion. Bars indicate the mean, and error bars indicate the standard deviation. *p < 0.05.

All results are shown in Figure 3. The anterior tensile forces exerted on the repair sutures at the inner and outer sides of the anterior segment of complete LM radial tears of the midbody were 13.7 ± 2.5 N/13.3 ± 3.2 N (mean ± standard deviation, inner/outer) at 30° flexion, 7.6 ± 3.7 N/7.3 ± 2.4 N at 60° flexion and 3.4 ± 2.0 N/3.5 ± 1.6 N at 90° flexion. The posterior tensile forces exerted on the repair sutures at the inner and outer sides of the posterior segment of complete LM radial tears of the midbody were 1.8 ± 1.5 N/1.5 ± 1.1 N at 30° flexion, 10.6 ± 4.8 N/9.7 ± 4.7 N at 60° flexion and 16.2 ± 3.9 N/16.4 ± 4.4 N at 90° flexion.

The tensile forces on the inner and outer sides of the complete LM radial tears in the anterior and posterior directions differed significantly from the knee flexion angles. At 30° flexion, the tensile forces on the inner and outer sides in the anterior direction were significantly greater than those in the posterior direction (*p* < 0.01). At 60° and 90° flexion, the forces in the posterior direction were significantly greater than those in the anterior direction (*p* = 0.02 and <0.01). The tensile forces on the repair sutures (in both the anterior and posterior directions) showed no significant differences between the inner and outer meniscal sides at 30°, 60° or 90° flexion.

## DISCUSSION

The most important finding of this study was that, under 5 N m valgus loading, the tensile force on the repair sutures in complete LM radial tears occurred anteriorly in the anterior segment at 30° flexion and posteriorly in the posterior segment at 90° flexion. Furthermore, the tensile force on the repair sutures in the anterior and posterior directions was not significantly different between the inner and outer meniscal sides at 30°, 60° or 90° flexion.

The tibiofemoral cartilage contact area in the lateral compartment of an intact knee is located in the anterior portion during extension and in the posterior portion during flexion [8]. The anterior horn of the intact LM moves 9.5 mm and the posterior horn 5.6 mm in an anteroposterior direction from 0° to 90° flexion in weight‐bearing, as measured by magnetic resonance imaging [32]. In addition, normal knee kinematics demonstrated medial pivot motion and bicondylar rollback motion from extension to flexion in a three‐dimensional in vivo motion analysis [28]. The lateral femoral condyle demonstrates consistent posterior translation throughout knee flexion in normal knees. The results of this study are consistent with the medial pivot and rollback motion relationship because the compressive loads reflect the converted tensile force, considering the knee meniscus function. Furthermore, these results are similar to those of a previous study that found load distribution and transmission through the LM in a complete radial tear of the midbody [16]. Although this study only evaluated LM radial tears of the midbody, we speculate that in terms of rehabilitation, loading at deep knee flexion angles should be avoided in meniscal posterior segment injuries compared with that at shallow flexion angles, whereas extension loading should be avoided in anterior segment injuries.

A previous study reported that the tensile force after pullout repair for medial meniscal posterior root tears under axial loads of 100–500 N at 30°, 60° and 90° flexion was 10–30 N [22]. Although the loading conditions and medial meniscus or LM differed, the results of this study are consistent with those of previous studies. For instance, another study reported that the tensile force acting on sutures in LM longitudinal tears under axial loads of 300 N with internal torsion at 30–90° flexion was 3.2–3.9 N [20]. Although longitudinal tears in the posterior segment did not separate to form a gap at the tear site under axial loading at 60° flexion in biomechanical studies of freshly frozen porcine knees, radial tears in the posterior segment did [24]. Although the loading conditions were different, LM radial tears resulted in greater tensile forces than longitudinal tears. The tensile properties of the tissue are different in the circumferential and radial directions. The tensile modulus in the circumferential direction is approximately 10‐fold higher than that in the radial direction [29]. Therefore, in contrast to longitudinal tears, early weight‐bearing after meniscal repair for LM radial tears should be avoided. Furthermore, the magnitude of the valgus load in this study was 5 Nm. The tensile force at the porcine meniscus is related to the femorotibial compression load [21]. The movement of the anterior horn of the LM was also greater when the knee was weight‐bearing relative to when it was non‐weight‐bearing [32]. Thus, the tensile force applied to the LM is expected to be higher when greater loads are applied.

The tensile force on the repair sutures did not differ significantly between the inner and outer sides of the meniscus. The resultant force on the meniscus of the longitudinal tear in the middle to posterior segments did not differ significantly between the inner and outer sides [26]. As the meniscus is a wedge structure, it was assumed that compressive forces from the femur were applied equally to the inner and outer sides. Therefore, it was assumed that the hoop structure converted the compressive forces equally into circumferential tensile forces on the inner and outer sides. Because the inner and outer sides of the meniscus are subjected to the same magnitude of tensile forces, it is desirable to suture both sides during meniscal repair. In this study, the distances of the repair sutures through the meniscus differed between the inner and outer sides. Therefore, the reason that the tensile force on the repair sutures did not differ between the inner and outer sides remains unclear, and further studies are required.

This study had some limitations. First, freshly frozen porcine knees were used in this study. There are anatomical differences between porcine and human knees, such as posterior tibial slope, absence of LM posterior root attachment, meniscal stiffness and extension lag [9, 11, 27]. Consequently, these tests were only performed at limited flexion angles and were not performed at full (0°) extension. However, porcine and human knees exhibit anatomical similarities [9, 18], and the porcine LM model has been used in several related biomechanical studies [7, 16, 17, 25]. Although using human cadavers may be more clinically relevant, there is a risk of degenerative changes to the meniscus that may not be obvious under direct visual evaluation, particularly in cadavers obtained from older people. Therefore, porcine knees were used in this study to reduce the influence of these quantitative variations. Second, we did not evaluate the peripheral meniscal gap under valgus loading. It was difficult to assess the peripheral gap in the porcine knees at a 5 N m valgus load using ultrasound because of the narrow joint space. Third, the measured tensile force was the combined force in the anteroposterior and extrusion directions, although the two load cells on the anterior and posterior sides were placed as parallel as possible on the same line to minimize the effect of the extrusion direction. Therefore, the values in this study may be less than the actual tensile force exerted on the repair sutures. Fourth, as this study did not evaluate the failure load after meniscus repair for LM radial tears, it is not known to what extent a load can be considered to exert a safe level of tensile force. Fifth, this study is a time zero model, the result did not account for any biological healing or muscle activity. Sixth, the loading condition in this study was only 5 N m valgus loading. This condition was determined by previous biomechanical study for LM radial tear [17, 19, 25]. Despite these limitations, the study provides valuable information on repaired LM tears to improve meniscal repair techniques and rehabilitation protocols.

## CONCLUSION

Tensile force is exerted on the repair sutures in complete LM radial tears of the midbody, anteriorly in the anterior segment at 30° flexion and posteriorly in the posterior segment at 90° flexion.

## AUTHOR CONTRIBUTIONS

*Conception and study design, testing, data acquisition, statistical analysis, literature review and manuscript writing*: Kodai Hamaoka. *Supervision*: Tomoaki Kamiya and Atsushi Teramoto. *Conception, study design and manuscript editing*: Kousuke Shiwaku. *Internal review*: Kazushi Horita, Yasutoshi Ikeda, Yohei Okada and Makoto Emori. *Conception and internal review*: Hidenori Otsubo.

## CONFLICT OF INTEREST STATEMENT

The authors declare no conflicts of interest.

## ETHICS STATEMENT

The study protocol did not require approval from the Institutional Review Board of our institute, because all porcine knee specimens were obtained from a butcher.

## Data Availability

Data are available from the corresponding author upon reasonable request.
